# Nervous System Injury and Neuroimaging of Zika Virus Infection

**DOI:** 10.3389/fneur.2018.00227

**Published:** 2018-04-23

**Authors:** Shanshan Wu, Yu Zeng, Alexander Lerner, Bo Gao, Meng Law

**Affiliations:** ^1^Department of Radiology, Affiliated Hospital of Guizhou Medical University, Guiyang, China; ^2^Department of Radiology, Yantai Yuhuangding Hospital, Yantai, China; ^3^Department of Radiology, Keck School of Medicine, University of Southern California, Los Angeles, CA, United States

**Keywords:** Zika virus, central nervous system infection, microcephaly, Guillain-Barre syndrome, neuroimaging

## Abstract

In 2016, World Health Organization announced Zika virus infection and its neurological sequalae are a public health emergency of global scope. Preliminary studies have confirmed a relationship between Zika virus infection and certain neurological disorders, including microcephaly and Guillain–Barre syndrome (GBS). The neuroimaging features of microcephaly secondary to Zika virus infection include calcifications at the junction of gray–white matter and subcortical white matter with associated cortical abnormalities, diminution of white matter, large ventricles with or without hydrocephalus, cortical malformations, hypoplasia of cerebellum and brainstem, and enlargement of cerebellomedullary cistern. Contrast enhancement of the cauda equine nerve roots is the typical neuroimaging finding of GBS associated with Zika virus. This review describes the nervous system disorders and associated imaging findings seen in Zika virus infection, with the aim to improve the understanding of this disease. Imaging plays a key role on accurate diagnosis and prognostic evaluation of this disease.

## Introduction

Zika virus was first isolated from the specimens of sentinel rhesus monkey in 1947 (1). The first human case of infection with Zika virus was reported in 1954 (1). However, at that time few suspected that Zika virus would later spread globally and cause a serious threat. Since 2015, the transmission of Zika virus has been reported in Brazil with subsequent spread to other regions including North America, with majority of cases still found in Brazil (2). An increase of the number of children born with microcephaly and Guillain-Barre syndrome (GBS) was also reported (1, 2). An increasing incidence of microcephaly and GBS in areas affected by Zika virus transmission was observed (2). Most researchers recognized that microcephaly and GBS might be associated with Zika virus infection (2). Understanding the imaging findings of microcephaly and GBS can contribute to early diagnosis of Zika virus infection. The purpose of this review is to summarize the imaging features of microcephaly and GBS in order to improve our understanding of these disorders associated with Zika virus.

### Transmission of Zika Virus

Zika virus is a member of the genus flavivirus of the flaviviridae family, which is mostly transmitted by *Aedes* species mosquito. Mosquitos become infected when they feed on the Zika virus carriers. The infected mosquitoes can spread the virus to humans by transmitting the virus through a bite when they feed. Multiple studies have shown that Zika virus may have a variety of other transmission routes. Zika virus can be transmitted from mother to fetus through placenta or transmitted to the newborn during delivery. Zika virus RNA has even been detected in breast milk (3); however, there are currently no reports of transmission of Zika virus through breast feeding. The virus is also reported to be transmitted *via* blood transfusion and sexual intercourse (4–6); however, these routes of transmission are rare. At present, Zika virus has been detected in the blood, urine, amniotic fluid, semen, saliva, cerebrospinal fluid (CSF), and even tears. The diversity of the transmission routs of Zika virus may be an important contributor to its rapid spread, while also increasing the difficulty of preventing and controlling this pathogen. Currently, there is no vaccine against Zika virus. Experimental studies have found that a unique interaction between Zika virus E protein residues increased its thermal stability, constituting a unique biochemical mechanism of Zika virus (6). This may help explain why the virus is stable enough to withstand harsh conditions such as urine, saliva, and semen. The structural stability of the virus makes it more adaptable, which may explain why it has the special ability to spread through sexual activity. These findings suggest that antibodies or pharmacologic agents that destabilize the structure of Zika virus may help reduce its ability to infect and limit its spread (7).

### Diagnosis of Zika Virus Infection

Zika virus infection is frequently subclinical, with only about 20% of infected patients demonstrating clinical symptoms (8). Zika virus infection is a self-limited disease, and the symptoms typically last for about 1 week. It is characterized by fever, maculo-papular rash, headache, conjunctivitis, arthralgia, sore throat, and muscle joint pain (8). Some infected patients may present with GBS, which mainly manifests as transient paralysis and varying degrees of sensory disturbance (9). Infection is difficult to diagnose because of Zika virus’s cross-reactivity with other flavi viruses, such as dengue virus (10). When the other flavi viruses are excluded, Zika virus can be diagnosed by detecting viral RNA using reverse transcriptase-polymerase chain reaction (RT-PCR) in serum of patients with symptoms or up to 1–3 days later (11). Zika virus can also be detected in saliva, urine, and semen (12, 13). The laboratory diagnosis of Zika virus infection includes RNA virus nucleic acid detection, virus-specific IgM, and neutralizing antibodies (11). Zika virus IgM antibodies can be detected by using serological tests, such as immunofluorescence and enzyme-linked immunosorbent assay (11), although Zika virus has extensive cross-reactivity with other flaviviruses. Serological testing is still an important diagnostic measure of Zika virus infection.

## Neuroimaging Findings of Zika Virus Infection

The nervous system damage caused by Zika virus infection is divided into two types: prenatal infection and postnatal infection (14). Prenatal infection can lead to microcephaly, cerebral calcification, cerebral cortical malformation, ventriculomegaly, corpus callosum hypoplasia, and subependymal cysts. Postnatal Zika virus infection may lead to severe neurological complications, including GBS, acute transverse myelitis, meningoencephalitis, and acute disseminated encephalomyelitis (15).

### Radiological Features of Microcephaly

#### Ultrasound

The first case of microcephaly related to Zika virus infection was reported in Brazil in October 2015 (1). Microcephaly is defined as neonatal head circumference ≥ 2 SD below the mean for gestational age, but in Brazil, the diagnostic criteria define this condition as neonatal head circumference ≥3 SD below the mean values (16). In March of 2016, the criteria adopted by the World Health Organization is that a head circumference at birth ≤31.7 cm for boys and 31.5 cm for girls (16). However, a fetus with multiple imaging findings of Zika virus infection, such as intracranial calcifications, may also have a normal head circumference. In this case, when we exclude all other possible causes, the possibility of a Zika virus infection should be considered. Recently, Aragao et al. (17) Research findings that there is may exist a disease specteum of Zika infection, and the specteum contains three degrees of severity: infants born with microcephaly, postnatal microcephaly, and the infants without microcephaly. This information could help to understand why some patients have only mild brain damage or even without microcephaly. Additional, Both placental insufficiency and fetal brain abnormalities can be detected by prenatal ultrasound and Doppler (18). In a lately preliminary study, 88 pregnant women were followed, and 72 of these women had a confirmed Zika infection, and only 42 had prenatal ultrasonography examinations during pregnancy (18). Fetal abnormalities were detected by Doppler ultrasonography in 12 women of 42 Zika virus-positive women, with or without microcephaly and intracranial calcifications (18). In a previous report, two pregnant women with fetal microcephaly possibly associated with the Zika virus were also noted to have the similar imaging features (19). Other imaging findings of infection include parenchymal atrophy, parenchymal calcification, ventriculomegaly, corpus callosum abnormalities, cerebellar, and brainstem hypoplasia, which can also be observed by ultrasound examination (20). In 2016, a case of an infected mother was reported who had a febrile illness with rash, Ultrasonography performed at 29 weeks of gestation revealed microcephaly with calcifications in the fetal brain and placenta; however, the examination did not identify abnormalities in early and mid-term pregnancy (21). The authors suggested that we need more appropriate imaging techniques to detect the lesions in the early stage.

#### Computed Tomography

**Figure 1:**
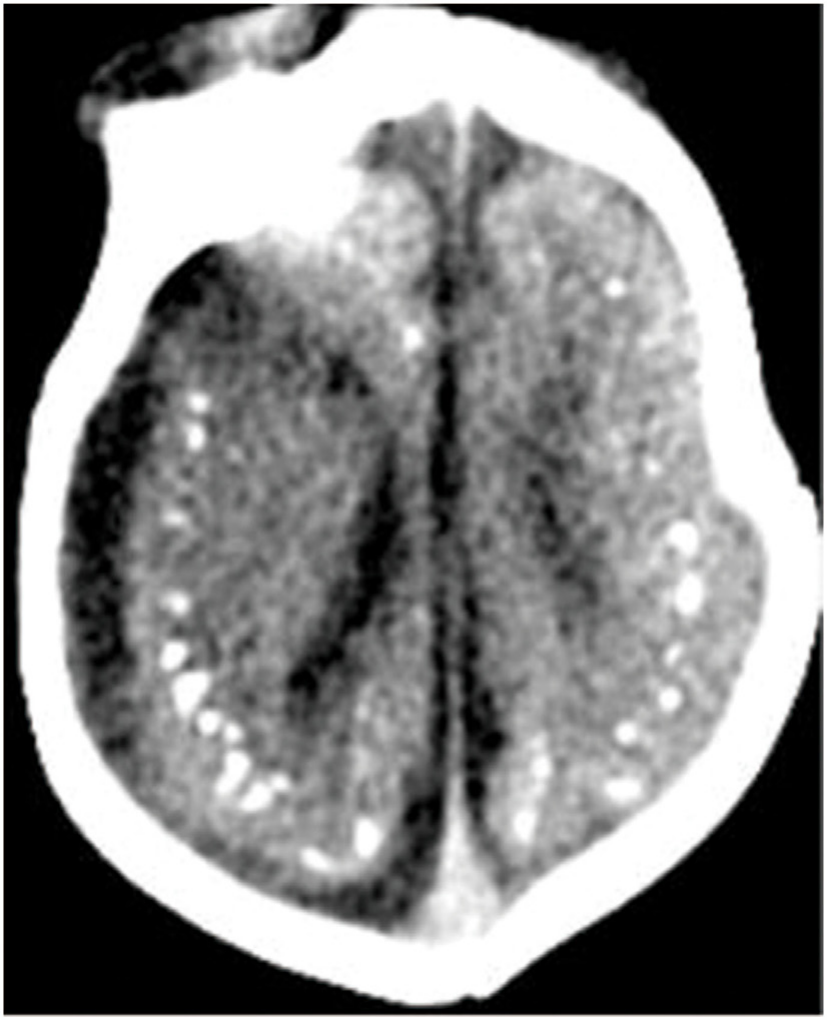
Axial CT image shows calcifications at the junction of the corticex and subcortical white matter, and parenchymal atrophy. All figures are courtesy of Lara Brandão M.D., Rio De Janeiro, Brazil.

Microcephaly is the principal characteristic of congenital infection during the early stages of pregnancy. A case series from Brazil in 2015 described CT and transfontanellar cranial ultrasound characteristics of Zika virus related microcephaly (1). Extensive calcifications of brain tissue can be observed in these cases, which are primarily found around the ventricles. Nearly one-third of cases also demonstrated cell migration abnormalities, such as lissencephaly and pachygyria. Ventricular enlargement secondary to cortical and subcortical atrophy was also observed (22). Hazin et al. (23) examined 23 cases of newborns with microcephaly using head CT and found intracranial calcifications, ventriculomegaly, cerebellar hypoplasia, and white-matter abnormalities. CT imaging is more sensitive for detection of calcifications than MRI. Intracranial calcifications are the most common imaging finding in Zika virus infection. The calcifications are primarily located in the periventricular brain tissue, at the junction of gray matter–white matter, subcortical white matter, thalamus, and basal ganglia. Other imaging findings include parenchymal atrophy, ventriculomegaly, malformations of abnormal cortical development, and corpus callosum abnormalities (23, 24) (Figure 1). It has also been reported that 3D virtual physical brain model of fetus with intrauterine infection of Zika virus may allow for a more intuitive way to observe the boundary of the giant ventricle, the corpus callosum hypoplasia, brain parenchymal atrophy, extensive brain calcification, and other associated findings (25, 26). In addition, CT scan also shows associated skull deformities, particularly using 3-dimensional reconstructions (14). We can also see severe craniofacial disproportion with depression of the frontal and parietal bones, cranial bone collapse, and a pointed appearance of the occipital and frontal regions. Moreover, on bone window and 3D reconstructions of head CT scans, the small fontanels can be seen. These findings are more likely secondary to the decrease of brain volume and reduced intracranial pressure.

#### Magnetic Resonance Imaging

**Figure 2:**
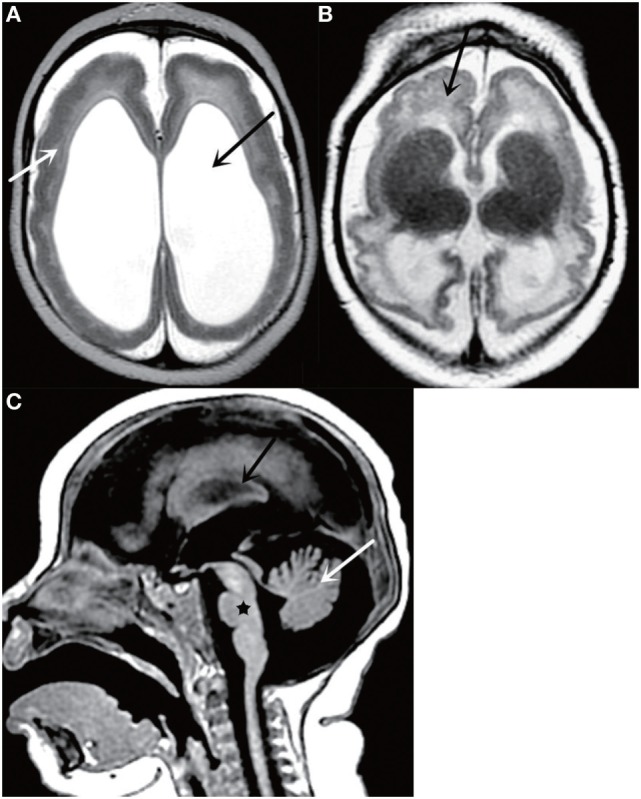
Axial T2-weighted MR image **(A)** shows a thickened cortex (white arrow) and ventriculomegaly (black arrow); Axial T2-weighted MR image **(B)** shows cortical malformations (black arrow); Sagittal T2-weighted MR image **(C)** shows cerebellar atrophy (white arrow), corpus callosum abnormalities (black arrow), and brainstem dysplasia (black star). All figures are courtesy of Lara Brandão M.D., Rio De Janeiro, Brazil.

Even though it is less sensitive for detection of calcifications, MRI can help detect other important findings such as polymicrogyria, laminar necrosis, and brainstem anomalies and avoid exposing infants to the ionizing radiation delivered by CT (Figure 2). In addition, MRI is much more sensitive for detecting cortical malformations and white matter abnormalities. de Fatima Vasco Aragao et al. (24) summarized the radiological findings observed on MRI scans in children with microcephaly and presumed congenital infection related to Zika virus. The characteristics on MRI images was similar to those observed on CT, which showed brain volume loss, ventricular enlargement due to white matter hypoplasia, associated with or without hydrocephalus, malformations of cortical development, calcifications, cerebellum and brainstem atrophy, enlarged cisterna magna, and enlarged anterior supratentorial subarachnoid space. Other imaging findings of Zika virus infection include polymicrogyria, corpus callosum abnormalities, and brainstem dysplasia. Additionally, occipital pseudocysts can also be seen resulting from intrauterine Zika virus infection (24, 27, 28).

MRI allowed for classification of decreased brain volume (mild, moderate, severe) and evaluation the development of the corpus callosum and the degree of myelination (29). Conventional MRI scanning sequences and susceptibility-weighted imaging showed that brain calcifications were predominantly located at the junction of gray matter and subcortical white matter. Previous reports indicated that calcifications were primarily located in periventricular tissue, which may be due to ventricular enlargement and decreased brain parenchyma, making the cortical and subcortical white matter calcifications appear periventricular (29). Therefore, MRI would be helpful to more accurately localize calcifications, although it may underestimate the incidence of calcifications.

### Radiological Features of GBS

The correlation between Zika and GBS was first reported in 2014 in French Polynesia (30). At present, the imaging findings of GBS have been reported. Brasil et al. (31) described a 24-year-old patient who had clinical manifestation of GBS, who had an MRI scan on the 13th day of the onset of the symptoms, and imaging showed no significant abnormality, with no further imaging examination obtained. Fontes et al. (32) reported the neural axis MRI findings in a patient who developed neurological symptoms of GBS. Brain MRI showed contrast enhancement of bilateral facial nerves and right trigeminal nerve. Lumbar spine MRI demonstrated contrast enhancement in conus medullaris, dorsal roots of cauda equina, and ventral nerve roots. T2WI images revealed increased signal intensity in the spinal ganglia bilaterally. All of these features were consistent with the prior cases of GBS, which contributed to the differential diagnosis from the complications of Zika virus infection. Although, like the first case reported above, there are some patients with clinical manifestation of CBS who may not have special MRI findings. So, the limited value of MRI in GBS should be known.

### Other Neurological Syndromes

The Zika virus infection can also cause acute myelitis, meningoencephalitis, and acute disseminated encephalomyelitis. On spine MRI, The abnormal signal of acute myelitis often involves more than three segments of spinal cord, and spinal cord may be enlarged due to edema. In the involved segments of the spinal cord, we may seen variable degrees and patterns of post-contrast enhancement. Mecharles et al. (33) reported a 15-year-old girl diagnosed with acute myelitis associated with Zika virus infection. Cervical, thoracic, and lumbar spine MRI showed enlargement of cervical and thoracic spinal cord and hyper-intensity in cervical spinal cord, which suggested edema, but conus medullaris and lumbar roots were normal. The viral load of Zika virus was detected by real-time reverse transcriptase PCR in the patient’s serum, urine, and CSF on the second day of symptom onset. Repeat MRI showed the degree of cervical cord edema was reduced after hormone therapy. The presence of Zika virus RNA in the CSF has confirmed the neurotropism of Zika virus. Besides, Carteaux et al. (34) described an 81-year-old man infected with Zika virus showing symptoms of meningoencephalitis such as fever and coma, whose brain MRI was consistent with meningoencephalitis. Fluid-attenuated inversion recovery images showed asymmetric hyper-intensities in subcortical white matter, diffusion-weighted sequences showed multiple punctuated hyper-intensities, in the right Rolandic fissure showed a slight hyper-intensity suggesting meningitis. Zika virus was detected by RT-PCR and was found in the samples of CSF. All of these findings supported that Zika virus was associated with meningoencephalitis. On brain MRI, acute disseminated encephalomyelitis shows multiple ill-defined, asymmetric lesions on the subcortical, deep white matter and deep gray matter nuclei. And it is usually showed homogenous enhancement in all lesions. In addition, diffusion restriction may be seen in the acute stage of the disease.

## Differential Diagnosis

### TORCH Syndrome

TORCH syndrome is a viral or protozoan infection of brain tissue causing encephalitis, occurring primarily in the embryo or during the delivery process, referred to as congenital intrauterine infection or congenital TORCH infection. The word TORCH is an English acronym for several pathogens causing brain infection, including *Toxoplasma* infection and other infectious agents, such as rubella virus, cytomegalovirus, and herpes simplex virus. Among them, Cytomegalovirus infection is most similar to the congenital Zika syndrome (35).

TORCH infected brain tissue mainly causes necrotizing encephalitis, especially prone to violations of the periventricular white matter, focal necrosis resulting in limitation of calcification. On CT scan, TORCH syndrome was characterized by multiple and scattered nodular calcifications in the brain parenchyma. The calcification caused by cytomegalovirus infection is usually located around the ventricle, while the calcification caused by *Toxoplasma gondii* infection usually lies on the periphery and can be scattered in the brain parenchyma. The calcification of herpes virus infection can occur very late after 3 years of age. However, Zika infection mainly occurs at the junction of cortex. In addition, TORCH syndrome can also affect other systems, such as involving the retina, can cause retinal dysplasia or retinitis. Cytomegalovirus infection can cause abnormal fetal brain development, such as pachygyria, polymicrogyria malformation lissencephaly. In general, given the different shapes, locations of intracranial calcifications in combination with other clinical and laboratory findings, these diseases may be differentiated from each other without difficulty.

### Tuberous Sclerosis

Tuberous sclerosis is a congenital, familial, and hereditary diseases. The clinical manifestations of sebaceous adenoma, epilepsy, and mental retardation in triad of features. The CT examination of the disease often presents with multiple intracranial calcification were the main manifestations of calcification after the age of two. Calcification is often located in subependymal or cerebral cortex, characterized by multiple nodular calcification in the subependymal zone. However, most of the patients with tuberous sclerosis have a skin sebaceous adenoma, or other characteristic lesions that are frequently detected simultaneously (36).

### Idiopathic Hypoparathyroidism

Idiopathic hypoparathyroidism may be familial or sporadic with unknown etiology, or may be secondary to autoimmune disease, and may occur alone or in combination with other autoimmune diseases. The head CT examination can show multiple calcifications in the brain parenchyma, usually diffusely distributed in the basal ganglia and thalamus, cerebellar dentate nucleus, in cerebral hemisphere cortex and corticomedullary junction area, with bilateral, usually symmetrical distribution and patchy, strip-like, crescent shaped, or punctate appearance. For the metabolic and endocrine disorders, the location and morphology of calcification are not characteristic. The diagnosis of hypoparathyroidism should be made in combination with clinical information, especially biochemical tests (37).

### Aicardi–Goutières Syndrome (AGS)

Aicardi–Goutières syndrome is a rare group of the nervous system and skin involvement in early-onset hereditary diseases. Its main clinical features include multiple intracranial calcifications, white matter lesions, CSF increased in chronic lymphocytic and frostbite like lesions. It was first reported by Aicardi and Goutieres in 1984. Intracranial calcification mainly occurred in more than 90% children with AGS, The location of calcifications is usually at the basal ganglia, periventricular white-matter, and dentate nuclei in AGS. However, congenital Zika infection usually at the corticomedullary junction. Additionally, the lack of abnormal cortical development in AGS may help to differentiate this disorder from congenital Zika infection (24).

### RNASET2-Related Leukodystrophy

Mutations in RNASET2 are associated with an autosomal recessive disorder characterized by early-onset severe developmental disorders, usually microcephaly, seizures, and sometimes hearing impairment. This disorder is caused by the mutations in RNASET2 gene, which have been hypothesized to result in accumulation of ribosomal RNA within neuronal lysosomes. In this disease, calcification is not very common, only a few patients were seen showing subtle spot calcification at the basal ganglia, periventricular, and deep white matter. In addition, cortical malformation has not been described. It is important to be aware of this condition, as it may be clinically and radiologically indistinguishable from congenital Zika infection (38) (Table 1).

**Table 1:** Differential diagnosis of brain abnormalities in Zika virus infection and other diseases.

Type of abnormality	Zika virus	TORCH	Tuberosa sclerosis	Idiopathic hypoparathyroidism	Aicardi–Goutières syndrome	RNASET2-related leukodystrophy
Calcification	Cortical-subcortical junction, punctate, dystrophic, linear, orcoarse pattern	Multiple and scattered nodular calcifications in the brain parenchyma	Nodular calcifications usually occurs after 2 years of age	Diffusely distributed bilateral distribution, usually symmetrical	Basal ganglia, periventricular white-matter, and dentate nuclei	Not very common, a few patient could seen subtle spot calcification at the basal ganglia, periventricular, and deep white matter
Microcephaly	Seen	May be seen	Absence	Absence	Seen	Seen
Ventriculomegaly	Seen	May be seen	Absence	Absence	May be seen	May be seen
Cerebellum	Developmental deformity	Hypoplasia may be seen	Normal	Normal	Normal	Normal
Cortical malformations	Seen	May be seen	Absence	Absence	Absence	Absence

## Conclusion

Neurologic manifestations of Zika virus infection are primarily seen as microcephaly and GBS. The imaging examinations including fetal ultrasound, CT, and MRI are quite helpful to diagnose and evaluate this disease. MRI can identify more associated abnormalities than CT and should be the first choice of investigation where available. Imaging may be very useful in providing evidence of congenital Zika syndrome and in improving our understanding of this disease. These imaging findings can alert radiologists to the possibility of congenital Zika virus infection in infants.

## Author Contributions

BG and ML designed the direction of the article and provided professional guidance. SW and YZ systematically collected, sorted out the relevant researches, and completed the manuscript. AL revised the manuscript’s professional issues and grammatical errors.

## Conflict of Interest Statement

The authors declare that the research was conducted in the absence of any commercial or financial relationships that could be construed as a potential conflict of interest. The reviewer MS and handling Editor declared their shared affiliation.
